# Olfactory tubercle stimulation alters odor preference behavior and recruits forebrain reward and motivational centers

**DOI:** 10.3389/fnbeh.2014.00081

**Published:** 2014-03-14

**Authors:** Brynn J. FitzGerald, Kara Richardson, Daniel W. Wesson

**Affiliations:** ^1^Department of Neurosciences, Case Western Reserve UniversityCleveland, OH, USA; ^2^Department of Biology, Case Western Reserve UniversityCleveland, OH, USA

**Keywords:** olfaction, olfactory cortex, self-stimulation, ventral striatum

## Abstract

Rodents show robust behavioral responses to odors, including strong preferences or aversions for certain odors. The neural mechanisms underlying the effects of odors on these behaviors in animals are not well understood. Here, we provide an initial proof-of-concept study into the role of the olfactory tubercle (OT), a structure with known anatomical connectivity with both brain reward and olfactory structures, in regulating odor-motivated behaviors. We implanted c57bl/6 male mice with an ipsilateral bipolar electrode into the OT to administer electric current and thereby yield gross activation of the OT. We confirmed that electrical stimulation of the OT was rewarding, with mice frequently self-administering stimulation on a fixed ratio schedule. In a separate experiment, mice were presented with either fox urine or peanut odors in a three-chamber preference test. In absence of OT stimulation, significant preference for the peanut odor chamber was observed which was abolished in the presence of OT stimulation. Perhaps providing a foundation for this modulation in behavior, we found that OT stimulation significantly increased the number of *c-Fos* positive neurons in not only the OT, but also in forebrain structures essential to motivated behaviors, including the nucleus accumbens and lateral septum. The present results support the notion that the OT is integral to the display of motivated behavior and possesses the capacity to modulate odor hedonics either by directly altering odor processing or perhaps by indirect actions on brain reward and motivation structures.

## Introduction

Odors have long been known to possess degrees of attractiveness or aversion (Locke and Grimm, 1949). These hedonics can either be innate from birth or in other cases, conditioned through learning. In both cases, perception of odors on the ends of the hedonic spectrum may elicit robust behavioral reactions. For instance, the bold odor of decaying meat elicits quite a repulsive reaction in humans whereas contrastingly, the sweet smell of freshly baked bread is in most cases pleasant. The neural mechanisms underlying these hedonic-driven behavioral responses are becoming increasingly known (e.g., Sullivan and Leon, 1987; Mennella and Garcia, 2000; Rolls et al., 2003; Stevenson and Repacholi, 2003; Sullivan, 2003; Bensafi et al., 2007; Grabenhorst et al., 2007; Baum, 2009; Doucette et al., 2011; Ferrero et al., 2011; Bensafi et al., 2012; Kass et al., 2013), yet major questions still remain.

Rodents are an excellent model for studying the neurobiological mechanisms of odor hedonics. From birth, and during early postnatal life, rats and mice display robust behavioral responses to odors, especially maternal odors to aid in maternal localization and feeding (Blass and Teicher, 1980; Sullivan, 2003; Logan et al., 2012). Most commonly studied in adult rodents, fearful responses (aversion, freezing/immobility, threat assessment) are reliably observed in response to predator odors (Blanchard et al., 2001; Wallace and Rosen, 2001; Takahashi et al., 2005; Ferrero et al., 2011). Thus rodents must possess a highly sophisticated system for the detection and response to odors.

The control of odor hedonic-driven behaviors likely requires not only a fully functional olfactory system to detect and discriminate the stimulus over background stimuli (for review see Wilson and Mainen, 2006; Wilson and Sullivan, 2011), but also the relay of this information into emotional and reward-related brain structures. The olfactory tubercle (OT) is an olfactory structure residing in the ventral striatum with large amounts of known anatomical connectivity into brain reward structures (Ikemoto, 2007; Wesson and Wilson, 2011). Due to this, we previously predicted that the OT serves a major role in regulating odor hedonics (Wesson and Wilson, 2011). Possible evidence for the regulation of rodent behavioral responses to odors by the OT was provided in a recent study by Agustín-Pavón et al. (2014). In the mentioned study, the authors created lesions containing a portion of the OT and observed that female mice with lesions displayed less attraction to male odors (Agustín-Pavón et al., 2014). This finding raises the interesting possibility that the OT regulates odor-hedonic behaviors either by means of its intrinsic rewarding properties (e.g., Prado-Alcalá and Wise, 1984; Ikemoto, 2003) and/or connectivity with reward and motivated behavior centers.

In the present study, we sought to further explore the role of the OT in odor-guided behaviors and the brain reward system in mice. To manipulate OT activity, we employed focal micro-stimulation of the OT using bipolar electrodes—a well-established method to probe principles of both olfactory and reward system activity (Freeman, 1960; Phillips and Mogenson, 1969; Prado-Alcalá and Wise, 1984; Mouly et al., 1985; Mouly and Holley, 1986; Wilson and Sullivan, 1990; Carlezon and Chartoff, 2007). As predicted based upon previous results in rats (Prado-Alcalá and Wise, 1984; Ikemoto, 2003), we found that mice self-administered current stimulation into the OT. Further, persistent automatic stimulation of the OT altered behavior in a three-chamber preference test. Finally, in separate groups of mice, we explored the recruitment of brain reward centers using the immediate early gene *c-Fos*. We found that OT stimulation not only recruited OT neurons focally, but also those of structures known connected to the OT—providing initial mechanistic insights into the likely importance of the OT to odor hedonics.

## Materials and methods

### Experimental subjects

Adult male c57bl/6 mice (2–4 months of age), bred and maintained within the Case Western Reserve University School of Medicine animal facility were used. Food and water were available *ad libitum* except during behavioral testing. All experiments were conducted in accordance with the guidelines of the National Institutes of Health and were approved by the Case Western Reserve University’s Institutional Animal Care Committee.

### Chronic stimulating electrode implantation surgery

A first cohort of mice (*n* = 8) were initially anesthetized with Isoflurane anesthesia (3.5–3% in 1 L/min O_2_) before being transferred and mounted into a stereotaxic frame where Isoflurane was further provided (3–1%). Core body temperature was maintained at 38°C with a hot water-filled heating pad. Upon confirmation of anesthesia depth, the head was shaved, cleaned with betadine and 70% EtOH, and a single injection of lidocaine (0.1 ml of 1% in H_2_O, S.C.) was administered within the future wound margin. A single incision was made from ~3 mm posterior of the nose along the midline to lambda and the skull surface cleaned with 3% H_2_O_2_. A single craniotomy (1 mm diameter) was created on the skull overlying the site of the OT for implantation of the stimulating electrode. The electrode consisted of 240 µm diameter stainless steel insulated wires (A-M Systems, Carlsborg, WA, USA) twisted together and connected by silver epoxy onto an Omnetics micro PS1 connector (Minneapolis, MN, USA). A micromanipulator was used to lower the bipolar electrode into the craniotomy and further into the site of the OT. The electrodes and plug were then cemented onto the skull by means of dental cement and the wound closed with Vetbond (3 M; St. Paul, MN, USA). Rimadyl (Carprofen, Pfizer animal health, 5 mg/kg, S.C.) was administered immediately following surgery and animals allowed to recover on the heating pad for >4 h. Rimadyl was administered daily for 5 days post-op. Food and water were available *ad libitum* except during behavioral procedures. All animals were singly-housed starting the day of implantation on a 12:12 h (light:dark) schedule with all behavioral procedures occurring during the light phase of the cycle (12:00:18:00 h). At least 5 days of recovery from surgery was allowed prior to any behavioral procedures. Following all behavioral procedures, mice were overdosed with urethane (3 mg/kg, I.P.) and transcardially perfused with 10 ml of 0.9% NaCl followed by 15 ml of 10% formalin and brains removed for histological verification of electrode sites.

### Acute olfactory tubercle (OT) stimulation

A separate cohort of 21 mice were anesthetized via urethane injection (1.0 mg/kg, I.P.) and mounted on a stereotaxic frame upon a water-filled heating pad (38°C) for acute OT stimulation. The basic surgical methods follow as described above for the chronic stimulating electrode implantation surgery, but with a few notable differences described herein. A stimulating bipolar electrode (same as described above) was lowered into the site of the OT. The stimulating electrode connector was then connected by a headstage via a motorized commutator to a Cygnus Technology SIU-91 isolated current source (Delaware Water Gap, PA, USA). Stimulated mice received 5 trials of current delivery (200 s train of bimodal, rectangular pulses, 50 ms in pulse width (i.e., 10 Hz), 100 µA in amplitude) at a 1 min inter-stimulus interval. Sham mice simply remained on the heating pad with the electrode in their OT for the same duration of time as the stimulated mice. Following which, the electrode was gently raised out of the brain and the animals transferred onto a heating pad for 90 min prior to transcardial perfusion as described above.

### Self-administration behavior testing

The self-stimulation chamber was made of acrylonitrile butadiene styrene (ABS) plastic and consisted of a 150 × 150 mm (W × L) floor bordered by 225 mm tall walls and an open celling. One wall was removable to allow insertion and extraction of the mouse from the testing chamber. Above the chamber was a video camera for recording behavioral events as well as a motorized commutator (Tucker Davis Technologies, Alachua, FL, USA) to allow the mice to be freely mobile but still connected to the stimulation tether. In the center of the chamber floor was a 10 × 25 mm piezo electric foil (“touch pad”; Parallax, Inc., Rocklin, CA, USA) for reception of paw presses. All self-administration testing was performed in a dark room with illumination enough to see the subject’s behavior provided by a single dim red light.

Digitization of paw presses and triggering of stimulation via the paw presses occurred by means of a Tucker Davis Technologies recording amplifier (RZ5) running custom code. A threshold was set for triggering of stimulation based upon an average touch pad voltage while mice freely explored the self-stimulation chamber during acclimation. During self-stimulation testing therefore, touch pad contact that crossed the threshold triggered the delivery of current stimulation (0.5 s train of bimodal, rectangular pulses, 50 ms in pulse width (i.e., 10 Hz), 100 µA in amplitude).

On 2 consecutive days mice were connected to the stimulation tether and allowed to freely explore the self-stimulation chamber (with stimulation triggering off) for 30 min of acclimation to the testing apparatus. Following, over the course of the next days, mice were again connected to the stimulation tether and allowed to explore the self-stimulation chamber for behavioral testing wherein contact with the touch pad triggered stimulation. Stimulation occurred on a fixed ratio 1 schedule. Importantly, touch pad contact must have been released in order for the mouse to receive the next stimulation upon contact. Mice were allowed access to stimulation in the self-stimulation chamber for up to 60 min each day throughout which the number of presses and the time of each press event were recorded.

### Three-chamber odor preference behavior testing

For a test of odor preferences, a 600 × 300 × 300 mm (length × width × height) clear acrylic chamber was divided into three equal zones with black markings. Above the chamber was a video camera for recording behavioral events as well as a motorized commutator (Tucker Davis Technologies) to allow the mice to be freely mobile but still connected to the stimulation tether. All testing was performed in a dark room with illumination enough to see the subject’s behavior provided by a single dim red light.

A single perforated dark plastic stimulus container (20 mm diameter × 20 mm tall) was placed on each end of the preference chamber for all testing. These stimulus containers were designed to allowing olfactory inspection of their contents but no distinct visual, somatosensory, or gustatory cues. On the first day, the mice were connected to the stimulation tether and allowed to freely explore the preference chamber (with stimulation off) for 30 min of acclimation to the testing chamber and clean odor-less stimulus containers. On the second and third days, the mice were again connected to the stimulation tether but this time the stimulus containers contained either a 1 × 10^−3^ dilution of fox urine^1^ placed on a cotton ball (100 µl fluid) or 3 g of crushed peanut. We predicted that these two different stimuli would elicit unique investigation behaviors (time spent/investigation) related to their emotional values (Takahashi et al., 2005) and thereby would provide a test as to whether or not stimulation of the OT would impact odor hedonic-related behaviors. The side of the preference chamber containing each stimulus was counterbalanced across all mice. On 1 day per mouse (counterbalanced across mice), current stimulation was provided throughout the entire duration of a daily session (continuous bimodal train, rectangular pulses, 50 ms in pulse width (i.e., 10 Hz), 100 µA in amplitude). On each day the testing lasted 500 s, throughout which the amount of time spent in each zone of the preference chamber and the number of zone crosses were recorded onto video. Videos were scored off-line by a single experimenter (K.R.) manually tallying zone crosses (defined by contact of all four paws across the divider line) and cumulative time based upon the video stopwatch.

### *c-Fos* immunohistochemistry and quantification

Alternate 40 µm coronal brain sections were acquired from mice which received the acute OT stimulation paradigm or sham controls using a sliding microtome. ≥5 sections/mouse spanning regions ~0.8–0.4 mm anterior to bregma were collected and left floating in 0.03% sodium azide in Tris-buffered saline (TBS, pH 7.4) until staining. *c-Fos* immunohistochemistry followed the methods of Kang et al. (2011b). First, the brain slices were rinsed in TBS and then quenched in a solution of 0.3% hydrogen peroxide in methanol. They were rinsed again in TBS, and then in 0.1% TX-100 in TBS. Following the rinses, the sections were blocked in 5.0% NDS in 0.1% TX-100 (Jackson ImmunoResearch, West Grove, PA, USA) for an hour and then incubated overnight at 4°C in the anti-*c-Fos* primary antibody (1:1000, Calbiochem, EMD Millipore, Billerica, MA, USA). A subset of slices in each run were used as a primary antibody control to ensure specificity of staining. The next day, the sections were rinsed in a diluting buffer, incubated in a secondary antibody (1:600, Jackson ImmunoResearch), rinsed again in 0.1% TX-100 in TBS, and incubated in an Avidin/Biotinylated enzyme Complex kit (Vector Laboratories, Inc., Burlingame, CA, USA). Finally, the sections were rinsed with 0.1% TX-100 in TBS, and incubated in a peroxidase substrate kit with diaminobenzidine (Vector Laboratories) and rinsed with ddH_2_O. Sections were then transferred onto slides and, after drying, cover slipped with Permount (Fisher Scientific, Pittsburgh, PA, USA).

*a priori* regions for analysis included the OT, ventral pallidum (VP), nucleus accumbens (NAc), lateral septum (LS), and caudate putamen (CPu). These regions were identified using known cytoarchitectural features (Paxinos and Franklin, 2000) and imaged at 20x magnification using a Leica microscope and a 3MP camera. Equal size (200 µm^2^) bounding boxes were overlaid upon the digital images for cell counting. The location of the bounding boxes were held constant across mice. Any cell bodies which touched the bounding box were excluded from counts. *c-Fos*+ cell bodies were manually identified and counted by a single observed based upon density of the 3,3′-diaminobenzidine (DAB) reaction versus background. Sections containing significant damage within the bounding box from the stimulating electrode were excluded from analysis and replaced with non-damaged sections. All steps including sectioning, staining, imaging, and quantification were completed in a group-counterbalanced order by a single experimenter blind to the experimental group of the tissue (B.F.).

### Electrode placement verification

**Figure 1:**
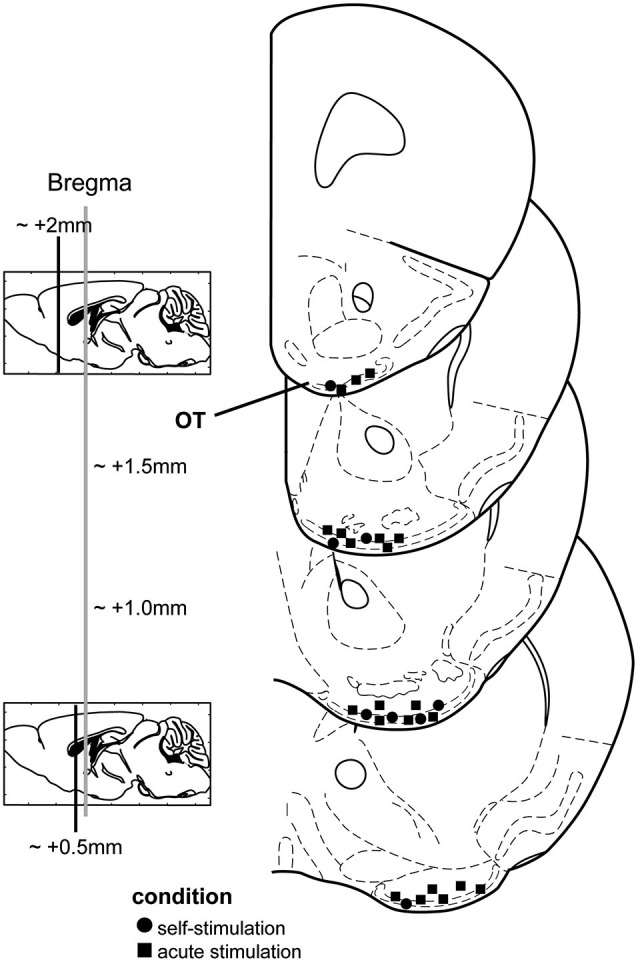
**Stimulation electrode locations from both chronic and acute stimulation experiments**. Mouse coronal stereotaxic panels displaying the location of stimulating electrode tips in the olfactory tubercle (OT) from mice self-administering stimulation (circles) and those which received acute stimulation (squares). Panels adapted from Paxinos and Franklin (2000).

All stimulation sites were verified by post-mortem histological examinations of slide-mounted 40 µm coronal brain sections stained with a 0.1% cresyl violet solution or in other cases, DAPI (4′,6-diamidino-2-phenylindole, Life Technologies, City State, USA). We defined an OT stimulation site as successful when the wires terminated within either layers i, ii, and/or iii of the OT (Figure 1). Electrode tip locations were verified by multiple-observers (B.F. and D.W.) with reference to a mouse brain atlas (Paxinos and Franklin, 2000). Data associated with sites outside of the OT were entirely excluded from this study.

### Data analysis

Behavioral data (time spent in preference zones, # touch pad presses, # zone crossings) were compared between conditions (stimulation on vs. stimulation off) with an ANOVA. The total number of *c-Fos*+ neurons was compared between conditions (stimulated vs. sham) and hemispheres (ipsilateral vs. contralateral) within brain regions specified also using an ANOVA. Data were analyzed in Origin 8.5 (Northampton, MA) with a significance level of *p* < 0.05. Values are reported as mean ± SEM unless otherwise noted.

## Results

**Figure 2:**
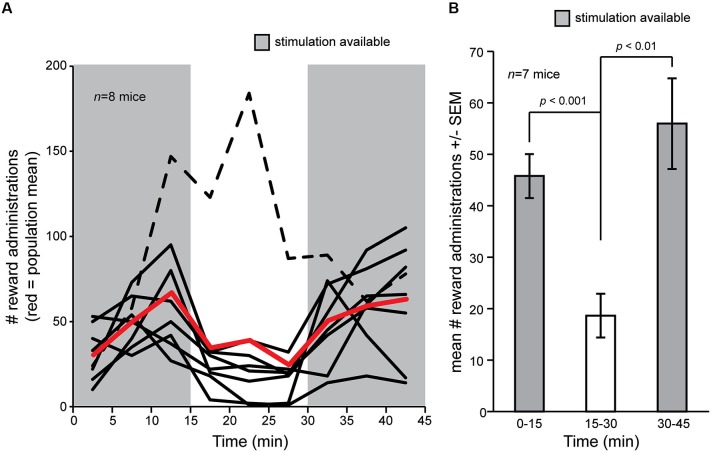
**Self-delivery of OT stimulation in mice. (A)** Mice (*n* = 8) were allowed to deliver current stimulation pulses for an initial block of 15 min, followed by a blank of 15 min wherein touch pad press did not delivery stimulation, and later a restoration block wherein again they were allowed to deliver current stimulation. Mice pressed more when stimulation was available (gray shaded regions) vs. when it was not, except for one mouse who strikingly increased the number of touch pad presses even though stimulation was not delivered (black dashed line). Red line = population mean, including outlier (dashed line). **(B)** Mean number of reward administrations from **(A)** in each 15 min block in the self-stimulation task, but with the outlier (dashed line) subject removed from the mean. *p*-values = ANOVA followed by Fisher’s PLSD.

We first asked whether OT electrical stimulation is rewarding in mice. To address this, we allowed a cohort of eight mice chronically implanted with bipolar stimulating electrodes into the OT to freely explore the self-stimulation chamber for 2 days wherein contact with a touch pad triggered OT stimulation (see Section Materials and Methods). Following, on the third day, mice were again placed into the self-stimulation chamber and numbers of touch pad presses recorded. Stimulation was allowed *ad libitum* over two blocks of 15 min, separated by a single block of 15 min wherein touch pad contact did not trigger stimulation. During the first 15 min block, mice readily pressed the touch pad (Figure 2A). The touch pad contacts were not resultant from random contact by the mice since turning off the stimulus in the middle 15 min block entailed a significant decrease in touch pad presses in all but one mouse (7/8, 87.5%) (Figures 2A, B) (*F*_(1,12)_ = 19.576, *p* = 0.0008). Reinstatement of the *ad libitum* reward delivery significantly restored touch pad presses in the final 15 min block (Figures 2A, B) (*F*_(1,12)_ = 13.786, *p* = 0.003). These data demonstrate that electrical stimulation of the OT is rewarding in mice.

**Figure 3:**
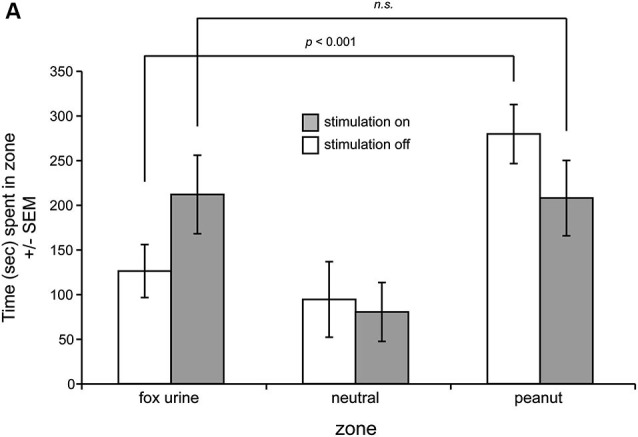
**OT stimulation alters odor-guided behaviors. (A)** Mice were allowed to explore a three-chamber odor preference chamber for 10 min with one side containing crushed peanuts (3 g) and the other fox urine (1:10 dilution) both contained within a perforated dark plastic container. On counterbalanced days, current stimulation was delivered throughout the entire duration of the session. On the day with stimulation off, mice spent significantly greater time in the peanut zone, whereas this was abolished with OT stimulation on. *n* = 6 mice. n.s. = not significant. *p*-values = ANOVA followed by Fisher’s PLSD.

We next investigated the influence of OT stimulation on odor preference behavior in the three-chamber preference test in the same mice. Notably, one mouse (Figure 2A, dashed line) displayed highly aberrant behavior from the group in the self-stimulation testing and was thus excluded from this preference experiment. A separate mouse did not explore the preference chamber and instead stayed immobile in a single zone—qualifying exclusion. Across the remaining six mice, all spent statistically similar time in both end zones in the presence of blank odor vials (*F*_(1,10)_ = 0.035, *p* = 0.855) (data not shown). This baseline data verifies that mice did not have a preference towards simply being on one side of the chamber vs. the other. Next, over 2 days, mice were tested for preference behavior among odorized zones, with OT stimulation being provided consecutively on one of those days. Mice received the stimulation on counterbalanced days (1/2 mice received it on day 1 and 1/2 on day 2), and thus in this design effects of OT stimulation on preference behavior at the population level can be considered independent of learning. No differences between these two groups were observed and thus their data were pooled together (*p* = 0.446, Kolmogorov-Smirnov test). We found that in the absence of OT stimulation, mice spent significantly greater time in the peanut zone of the chamber vs. either the neutral (*F*_(1,10)_ = 11.901, *p* = 0.0062) or the fox urine zones (*F*_(1,10)_ = 11.882, *p* = 0.0063) (Figure 3). OT stimulation strikingly abolished this difference, with statistically indistinguishable time spent in both the fox and peanut zones (*F*_(1,10)_ = 0.004, *p* = 0.949) (Figure 3). No effect of OT stimulation was observed on the number of side crossings in the preference apparatus (*F*_(1,10)_ = 0.235, *p* = 0.639) (data not shown), suggesting that the effect of OT stimulation on the display of behavior in the odor preference task was independent of stimulation influencing gross locomotor activity.

**Figure 4:**
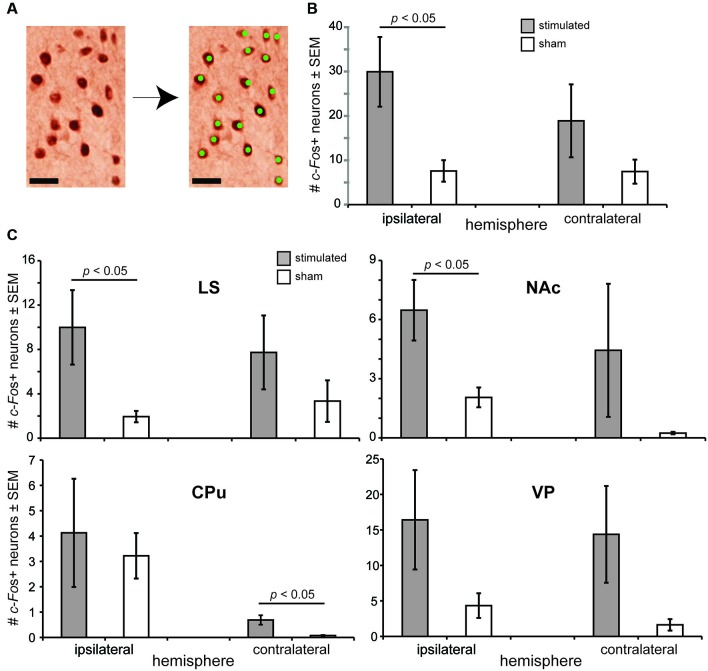
**OT stimulation recruits forebrain structures involved in motivated behaviors. (A)** Photomicrograph from an OT tissue sample stained for *c-Fos* (left) and the same micrograph following detection of *c-Fos*+ neurons. As illustrated, only neurons with intense enough reaction/staining were applied towards *c-Fos* counts (green dots). Scale bar = 20 µm. **(B)** OT stimulation elicits a significant increase in *c-Fos*+ neurons in the ipsilateral OT. *c-Fos*+ neuron counts were quantified within a 200 µm^2^ bounding box. **(C)** Effects of OT stimulation on the number of *c-Fos*+ neurons in forebrain reward and motivated behavior centers, including the NAc (nucleus accumbens), CPu (caudate putamen), LS (lateral septum), and VP (ventral pallidum). *n* = 13 mice stimulated, 8 mice sham for all structures, ≥5 sections/structure. *c-Fos*+ neuron counts were quantified within a 200 µm^2^ bounding box. *p*-values = ANOVA followed by Fisher’s PLSD.

Based upon the above behavioral findings demonstrating that OT stimulation influences odor-driven behavioral responses, we next sought to test the mechanisms whereby OT stimulation may alter hedonic-related behaviors. We predicted based upon known anatomical connectivity between the OT and brain reward structures (Ikemoto, 2007), that OT stimulation recruits forebrain structures necessary for reward (Koob and Le Moal, 2001; Berridge, 2003; Ikemoto, 2007). Therefore, a separate cohort of anesthetized mice received OT stimulation (*n* = 13) or sham OT stimulation (*n* = 8) in a paradigm mimicking that received while awake (see Section Materials and Methods) and later, their brains probed by means of immunohistochemistry for levels of *c-Fos* expression (Sagar et al., 1988; Figure 4A). The use of anesthetized mice for this analysis was advantageous to ensure changes in *c-Fos* expression were directly due to OT stimulation, vs. extraneous influences of OT stimulation upon behavior.

Confirming the physiological potency of the stimulation paradigm, we found that OT stimulated mice had a significantly greater number of *c-Fos*+ neurons in their ipsilateral OT than sham treated mice (*F*_(1,19)_ = 4.679, *p* = 0.0435) (Figure 4B). No group effect was observed when comparing between contralateral OT hemispheres (*F*_(1,19)_ = 1.157, *p* = 0.296) (Figure 4B). Looking beyond the OT, we found that mice receiving OT stimulation had a significantly greater number of *c-Fos*+ neurons compared to sham mice in their LS (*F*_(1,19)_ = 4.852, *p* = 0.040) and NAc (*F*_(1,19)_ = 4.828, *p* = 0.0406), but not their CPu (*F*_(1,19)_ = 0.032, *p* = 0.861) nor VP (*F*_(1,19)_ = 1.793, *p* = 0.196) (ipsilateral vs. ipsilateral) (Figure 4C). Across all of these structures, only in the CPu did the number of *c-Fos*+ neurons in the contralateral hemisphere significantly differ between stimulated and sham groups (*F*_(1,19)_ = 6.09, *p* = 0.023) (Figure 4C). No group effect of stimulation was observed between the contralateral hemispheres in the LS (*F*_(1,19)_ = 0.943, *p* = 0.344), NAc (*F*_(1,19)_ = 0.931, *p* = 0.347), or the VP (*F*_(1,19)_ = 2.179, *p* = 0.156). In all structures analyzed, the number of *c-Fos*+ neurons was statistically similar comparing between the ipsilateral to contralateral hemispheres (*p* > 0.05). For a reason we are unaware of, perhaps related to damage of near-by electrode insertion, sham mice displayed significant increases in the number of *c-Fos*+ neurons between their contralateral and ipsilateral hemispheres in both the CPu (*F*_(1,14)_ = 15.067, *p* = 0.0017) and NAc (*F*_(1,14)_ = 12.684, *p* = 0.0031). Taken together, these results suggest that the influence of OT stimulation on motivated and odor-driven behaviors may occur via connectivity between the OT and the NAc, LS, and possibly CPu.

## Discussion

In this study we provide an initial proof-of-concept exploration into the role of the OT, a structure with known anatomical connectivity with both brain reward and olfactory structures, in regulating odor-motivated behaviors and their possible mechanisms. We confirm that electrical stimulation of the OT was rewarding (Prado-Alcalá and Wise, 1984), in this case in mice, and possessed the capacity to alter odor-directed preference behaviors. In separate experiments we also found that OT stimulation significantly increased the number of *c-Fos* positive neurons in not only the OT, but also in forebrain structures essential to motivated behaviors, including the NAc and LS. The present results support the notion that the OT is integral to motivated behaviors and likely involved in odor hedonics.

### Influence of the olfactory tubercle (OT) on reward and odor-guided behaviors

In the present study we found that mice self-administered electrical current into the OT. This finding is analogous and complimentary to a much earlier finding by Prado-Alcalá and Wise (1984) also one by Ikemoto demonstrating that rats readily self-administer cocaine into the OT and further that cocaine infusions into the OT are sufficient for the development of a conditioned place preference (Ikemoto, 2003). Our self-administration experiments in the present study were not designed to be interpreted as novel in theory by any means but instead to reinforce the concept that the OT is instrumental in driving reward-related behaviors (Koob et al., 1978; Prado-Alcalá and Wise, 1984; Alheid and Heimer, 1988; Heimer, 2003; Ikemoto, 2003). Reward system projections into the OT include the rostral linear nucleus of the ventral tegmental area (Del-Fava et al., 2007), the medial forebrain bundle (Gaykema et al., 1990), the NAc (Zahm and Heimer, 1993), and the substantia nigra (Fallon et al., 1978). Thus, possibly due to both intrinsic (DAergic receptor expression (Li and Kuzhikandathil, 2012)) and extrinsic factors (inter-network connectivity), the OT appears capable of eliciting motivated behaviors. Exploring possible mechanisms of connectivity between the OT and the LS and CPu, as suggested herein, will be important in furthering our understanding of the OTs’ rewarding properties.

In addition to being connected with reward-related structures, the OT also receives dense innervation from secondary neurons in the olfactory bulb (e.g., White, 1965; Scott et al., 1980; Schwob and Price, 1984; Nagayama et al., 2010; Kang et al., 2011a) and consequently represents and processes odors in a manner similar to the primary (piriform) olfactory cortex (Payton et al., 2012). A recent study reported that lesions of the anteriomedial ventral striato-pallidum complex (including the OT) altered preferences of female mice for male sociosexual odors (Agustín-Pavón et al., 2014). This finding posed the important question as to whether or not manipulation of OT activity alone might be sufficient to alter odor guided behavior? Our present results support and extend the previous finding by Agustín-Pavón et al. (2014) by demonstrating that electrical manipulation of OT activity, specifically, possesses the capacity to modulate odor-guided behaviors. The use of electrical stimulation to modulate and explore principles of olfactory system function and olfactory behaviors is well established (Mouly et al., 1985; Mouly and Holley, 1986; Wilson and Sullivan, 1990) and thus we employed that method herein for bulk modulation of OT activity. Using this, we found that stimulation of the OT abolished preference for peanut odor vs. fox urine odor. Notably, using this gross odor preference behavior to test the OTs involvement in olfaction is a considerably insensitive assay and thus additional studies will be needed employing more precise olfactory read-outs to explore unique and specific aspects of olfactory behavior under control by the OT. Indeed, as they stand, our results demonstrate the role for the OT in modulating odor-guided behaviors, but not specifically modulating olfactory perception. That said, our results from the odor preference assay do support the idea that OT local and/or inter-regional processing modulates odor-guided behaviors if not at least in an indirect manner. This indirect mechanism could be by shaping motivation and/or the reward features of the odors (e.g., via modulation of structures identified in Figure 4). Directly, OT stimulation might perturb basic aspects of odor processing known to occur in the OT (Wesson and Wilson, 2010; Payton et al., 2012; Carlson et al., 2014) and thus alter odor perception more specifically. We predict that the effects observed in the present paper stem form a combination of both direct and indirect impacts of OT activity on behavior.

### Possible neural substrates whereby olfactory tubercle (OT) affects behavior

In the present study, we found that moderate electrical stimulation of the OT resulted in significant recruitment of neurons within brain structures believed essential to motivated behaviors (Koob and Le Moal, 2001; Berridge, 2003; Ikemoto, 2007), including the NAc and LS. Notably, this list is not exhaustive in that we did not analyze every possible motivated behavior center, but instead approached this study with an *a priori* set of forebrain regions. Likely analyzing additional brain structures, especially the ventral tegmental area and orbitofrontal cortex, would provide additional evidence for the functional interconnectedness of the OT (Barbas, 1993; Illig, 2005; Del-Fava et al., 2007). Additionally, the medial vs. lateral aspects of the OT are hypothesized differentially involved in olfactory and reward-related behaviors (Josephson et al., 1997; Ikemoto, 2007) and thus, it is possible that our grouping of OT stimulation sites spanning the entire OT overlooked more subtle aspects of neuronal output which otherwise might be observed if one were to employ regionally-restricted stimulation.

It is interesting to speculate upon why OT stimulation did not more strongly activate structures like the VP which also hold strong interconnectedness with the OT (Millhouse, 1986). While the number of *c-Fos*+ neurons was increased in this structure, this was not significant. It is possible that in the majority of animals bulk OT stimulation was insufficient to recruit distinct sub-populations of neurons innervating the VP and thus VP activation was minimal. Indeed, populations of VP projecting neurons from the OT are GABAergic (Meyer et al., 1989; Hsieh and Puche, 2013) and thus the effects of OT stimulation on the VP would be inhibitory. While the present study yields novel evidence upon the effects of gross activation of the OT, employing more precise stimulation methods, including genetically guided cell-specific methods, will be needed to more closely address mechanisms of OT connectivity.

## Conflict of interest statement

The authors declare that the research was conducted in the absence of any commercial or financial relationships that could be construed as a potential conflict of interest.

## Footnotes

^1^Predatorpee.com
